# Five-year follow-up with the incidence of cardiovascular adverse events after prescribing single-pill combination of antihypertensive drugs: retrospective cohort study

**DOI:** 10.1186/s40780-025-00510-w

**Published:** 2025-11-18

**Authors:** Misa Matsumura, Toshiaki Nakamura, Kaori Kadoyama, Ryoichi Yano, Kazunori Iwanaga, Kaori Imanishi, Kaori Yamanishi, Ichiro Nakakura, Yoshiko Une, Tsutomu Nakamura

**Affiliations:** 1 Education and Research Center for Clinical Pharmacy, Faculty of Pharmacy, Osaka Medical and Pharmaceutical University, 4-20-1 Nasahara, Takatsuki, 569-1094 Japan; 2 Department of Pharmacy, National Cerebral and Cardiovascular Center, 6-1 Kishibe-Shinmachi, Suita, 564-8565 Japan

**Keywords:** Hypertension, Fixed-dose combination, Adherence, Adverse events, Retrospective study

## Abstract

**Background:**

This study aimed to investigate whether medication adherence was changed by the prescription of a single-pill combination (SPC) of antihypertensive drugs and whether it was associated with the incidence of major adverse cardiovascular events (MACE) at the 5-year follow-up.

**Methods:**

The medical records of 707 patients whose medication adherence could be assessed at least one year before and after the date SPC was first prescribed were retrospectively reviewed. The patients’ baseline characteristics included sex, age, medical and medication history within the past year (MACE, antidiabetic drugs, and antihyperlipidemic drugs), and polypharmacy (six or more drugs taken orally). Medication adherence was evaluated as the proportion of days covered, and the patients were divided into poor adherence (*n* = 28) and good adherence (*n* = 679) groups. Fisher’s exact test and the Mann-Whitney U test were used to examine the differences in variables between the groups. Kaplan–Meier survival analysis was performed to assess time-to-event data, with group differences evaluated using the log-rank test and Cox proportional hazards models adjusted for baseline variables.

**Results:**

No significant differences were observed in the patients’ baseline characteristics between the good and poor adherence groups. During the first year after the initiation of prescribing the SPC of antihypertensive drugs, the percentage of patients who developed MACE was higher in the poor adherence group than in the good adherence group (21.4% and 11.8%, respectively), but the difference was not statistically significant (*p* = 0.137). No significant difference in the cumulative incidence of MACE during the 5-year observation period was observed between the poor and good adherence groups (*p* = 0.199). In the Cox proportional hazards analysis, 75 years of age or older, the occurrence of MACE within the past year, and polypharmacy were associated with increased risk of 5-year MACE compared to the references (adjusted hazard ratios 1.42 [95% Confidence Intervals: 1.10–1.83], *p* = 0.007; 0.72 [0.55–0.93], *p* = 0.011; and 1.36 [1.01–1.83], *p* = 0.044, respectively).

**Conclusions:**

The present findings demonstrated that age 75 or older age, history of MACE, and polypharmacy were significant risk factors for MACE after the prescription of antihypertensive SPC.

## Introduction

Cerebrovascular and heart diseases are among the leading causes of death in Japanese people [1], and appropriate control of blood pressure is very important to prevent the onset and worsening of major adverse cardiovascular events (MACE), thereby reducing the risk of death [2]. When a single drug is not sufficiently effective in treating hypertension, two or more drugs with different mechanisms of action may be used in combination [3]. In the 2024 European Society of Cardiology guidelines for the management of elevated blood pressure and hypertension, the initial dual treatment combinations comprised an angiotensin-converting enzyme inhibitor or angiotensin receptor blocker with either a dihydropyridine calcium channel blocker or a thiazide/thiazide-like diuretic [4]. This increases the number of medications taken, which may place a greater burden on patients. Some meta-analyses have revealed that single-pill combinations (SPCs) of two or more antihypertensive agents in a single pill provide greater benefits than the corresponding free-drug components administered separately [5, 6], and similar results have been reported in Taiwan [7], Korea [8], Europe, India [9], and Canada [10]. While the use of SPCs reduces the number of medications to be taken, which is expected to improve patient adherence to medication and lead to better blood pressure control, it has been reported that clinical risk factors do not always significantly improve [11].

As mentioned above, hypertension is one of the factors causing the incidence of MACE, and poor adherence and persistence to blood pressure-lowering agents lead to an increased risk of morbidity and mortality. A retrospective claims database analysis conducted by Tung et al. revealed that MACE-free survival and medication adherence were improved in patients taking a fixed-dose combination of valsartan/amlodipine compared with those taking a free combination regimen of angiotensin receptor blockers and calcium channel blockers at a 15-month follow-up [12]. However, real-world clinical data showing an association between medication adherence after the initiation of antihypertensive SPCs and the incidence of MACE remain obscure. Factors such as diabetes, dyslipidemia, obesity, and a family history of cerebrovascular disease or lifestyle-related diseases are thought to increase the risk of developing MACE.

In the present study, in patients undergoing drug therapy for hypertension, the change in medication adherence after starting the prescription of SPCs was evaluated, and the association between medication adherence to antihypertensive drugs and the incidence of MACE was also investigated.

## Patients and methods

### Patient selection and study protocol

The National Cerebral and Cardiovascular Center (NCVC) in Japan is a part of the National Center for Advanced and Specialized Medical Care and provides advanced and medical care for patients with severe or complex cardiovascular and cerebrovascular diseases [13]. Of the 2435 outpatients who were prescribed SPC for antihypertensive drugs at the NCVC from January 2012 to December 2018, we retrospectively reviewed the medical records of 707 patients whose medication adherence could be assessed at least one year before and after the date SPC was first prescribed. The SPC of the antihypertensive drugs used in this study and the number of patients are summarized in Table 1. This study was conducted according to the principles of the Declaration of Helsinki. The study protocol was approved by the local ethics committees of the NCVC (Institutional Review Board number, M30-164) and Osaka Medical and Pharmaceutical University (approval no. 2024 − 244). This was a retrospective cohort study and informed consent was obtained via the opt-out method.

**Table 1:** Single-pill combination (SPC) and corresponding single drug components

SPC of antihypertensive drugs	Single drug components	Number of patients
Aimix^®^ combination tablets	Irbesartan/Amlodipine	152
Atedio^®^ combination tablets	Valsartan/Cilnidipine	2
Xforge^®^ combination tablets	Valsartan/Amlodipine	33
Zacras^®^ combination tablets	Azilsartan/Amlodipine	58
Micamlo^®^ combination tablets Teramuro^®^ combination tablets	Telmisartan/Amlodipine	158
Unisia^®^ combination tablets	Candesartan/Amlodipine	77
Rezaltas^®^ combination tablets	Olmesartan/Azelnidipine	54
Irtra^®^ combination tablets	Irbesartan/Trichlormethiazide	5
Ecard^®^ combination tablets	Candesartan/Hydrochlorothiazide	23
Co-DIO^®^ combination tablets	Valsartan/Hydrochlorothiazide	9
Preminent^®^ combination tablets Losarhyd^®^ combination tablets	Losartan potassium/Hydrochlorothiazide	48
Micombi^®^ combination tablets Telthia^®^ combination tablets	Telmisartan/Hydrochlorothiazide	65
Caduet^®^ combination tablets	Amlodipine/Atorvastatin	82
Micatrio^®^ combination tablets	Amlodipine/Telmisartan/Hydrochlorothiazide	2

SPC, single-pill combination

The number of patients includes double counting

### Clinical parameters, data collection, and assessment

All data were collected using an electronic medical records system. We assessed the following patient clinical characteristics at the time of SPC prescription of antihypertensive drugs: sex, age (75years of age or older), medical and medication history within the past year (MACE, adherence, antiplatelets, antidiabetic drugs and antihyperlipidemic drugs), and polypharmacy (six or more oral drugs taken). Emergency medications were excluded when the number of oral medications used was counted.

### Major adverse cardiovascular events (MACE)

MACE was defined as a subcategory or disease classified under the International Classification of Diseases, 10th version (ICD-10) (2013 edition), which included: unstable “angina pectoris” (I20), “acute myocardial infarction” (I21), “subsequent myocardial infarction” (I22), “sudden cardiac death, so described” (I46.1), “heart failure” (I50), “subarachnoid hemorrhage” (I60), “intracerebral hemorrhage” (I61), and “cerebral infarction” (I63). The time to onset of MACE was defined as the time to the first onset within five years from the date when the SPC of antihypertensive drugs was first prescribed.

### Evaluation of medication adherence

Medication adherence was evaluated as the proportion of days covered (PDC), which was estimated as the number of days the drug was prescribed divided by the number of days in the interval between outpatient visits. The PDC was calculated for the antihypertensive drugs administered orally, including angiotensin converting enzymeinhibitors, angiotensin receptor blockers, aldosterone antagonists, calcium channel blockers, thiazide/thiazide-like diuretics, *β*-blockers, and *α*_1_*β*-blockers, by each patient, and the average values were used as the PDC for each patient. Concerning medication adherence, Baumgartner et al. conducted a meta-analysis of six studies that assessed clinical outcomes linked to medication adherence rates in chronic disease states by calculating the medication possession ratio, PDC, or both and suggested the appropriateness of the conventional value of 0.8 by verifying the medication adherence threshold in relation to a targeted clinical outcome [15]. In the present study, the patients were divided into two groups at the time of SPC prescription of antihypertensive drugs: those with a PDC within the past year of less than 0.8 (poor adherence group, *n* = 28) and those with a PDC within the past year of 0.8 or higher (good adherence group, *n* = 679).

### Statistical analysis

Fisher’s exact test was used to examine the differences in categorical variables between groups. Continuous data were compared using the Mann-Whitney U test. Univariable and multivariable Cox regression models adjusted for baseline variables, along with Kaplan-Meier survival analysis and the log-rank test, were used to assess time-to-event data, with group differences. In the Kaplan-Meier analysis, patients were censored when they were lost to follow-up for more than 3 months or when MACE occurred. All statistical analyses were performed using EZR (Saitama Medical Center, Jichi Medical University, Saitama, Japan), which is a graphical user interface for R (R Foundation for Statistical Computing, Vienna, Austria) [14]. Results with two-tailed p-values less than 0.05 were considered statistically significant.

## Results

### Patients’ backgrounds

The patient demographic data and medical and medication histories are shown in Table 2. This study included a total of 707 patients (428 males [60.5%] and 279 females [39.5%]) for whom SPC of antihypertensive drugs were prescribed. No significant differences were observed between the good and poor adherence groups in terms of age, sex, medical and medication histories within the past year, or polypharmacy status.

**Table 2:** Patients’ background at the beginning of SPCs and the observation period

Variable	Poor medication adherence PDC < 0.8	Good medication adherence PDC ≥ 0.8	*p*
	(*n* = 28)	(*n* = 679)	
Male Female	21 7	407 272	0.119
Age (years) <75 75≤	69.0 [64.5–74.3] 21 7	72.0 [63.0–78.0] 422 257	0.296 0.231
History of MACE within the past year			
Yes No	18 10	438 241	1.000
Medications history within the past year			
Antidiabetic drugs			
Yes No	6 22	138 541	0.814
Antihyperlipidemic drugs			
Yes No	14 14	399 280	0.435
Antiplatelet drugs			
Yes No	19 9	389 290	0.331
Number of concomitant oral drugs within the past year			
< 6 6≤	20 8	433 246	0.547
Observation period (days)	325.5 [188.3–773.5]	497.0 [189.0–1256.5]	0.111

Data are expressed as median and interquartile range for continuous values and number of subjects for categorical values

Categorical data were analyzed using the Fisher’s exact test

Continuous data were compared between the good and poor adherence groups using the Mann-Whitney U test

Abbreviations: SPC, single-pill combination; PDC, proportion of days covered; MACE, major adverse cardiovascular event

### Incidence of MACE

**Fig. 1:**
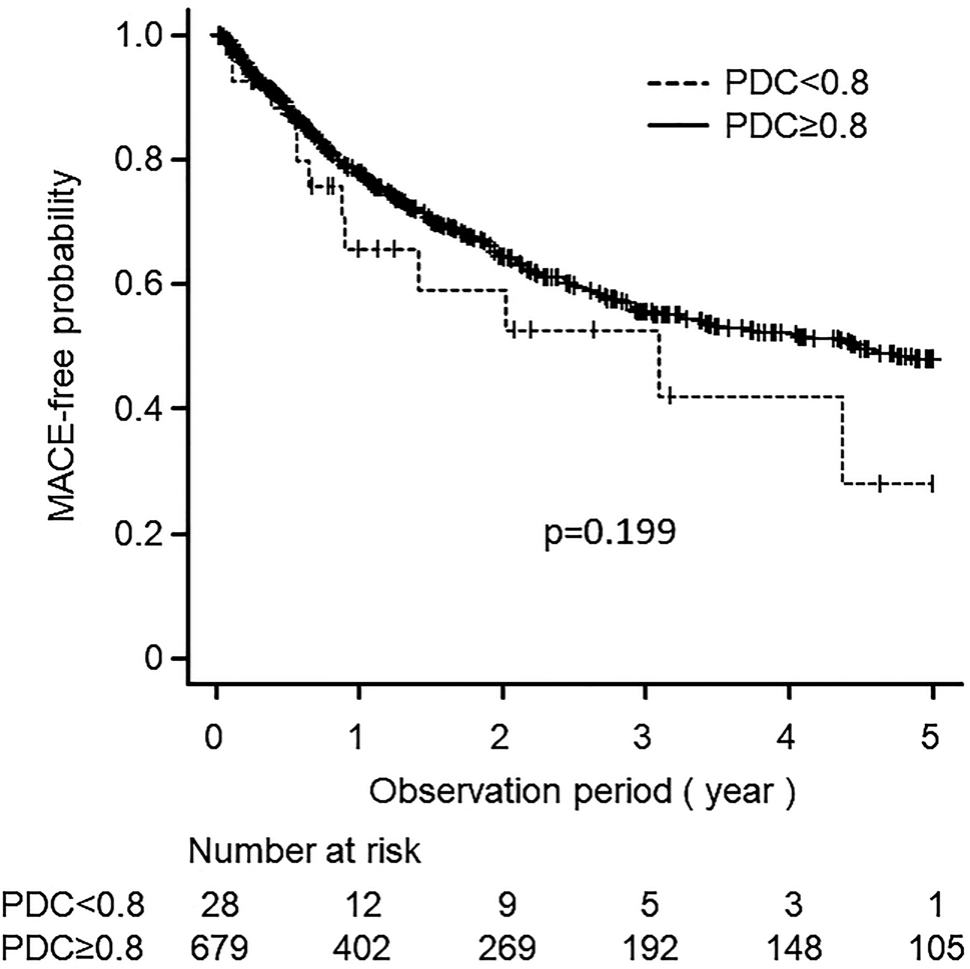
Cumulative MACE-free probability during 5-year follow-up for patients receiving SPC-based antihypertensive therapy. The patients were divided into two groups: those with a PDC of less than 0.8 (poor adherence group, *n* = 28) and those with a PDC of 0.8 or higher (good adherence group, *n* = 679). Dashed and solid lines represent poor and good medication adherence groups, respectively. MACE, major adverse cardiovascular events; SPC, single-pill combination; PDC, proportion of days covered

During the first year after prescribing the SPC of antihypertensive drugs, 86 (12.2%) of the 707 patients experienced MACE, and the percentage of patients who developed MACE was higher in the poor adherence group than in the good adherence group (21.4% [6/28] and 11.8% [80/679], respectively); however, the difference was not statistically significant (*p* = 0.137). Kaplan–Meier analysis showed a cumulative incidence of MACE during the 5-year observation period of 42.9% [12/28] and 37.0% [251/679] in the poor and good adherence groups, respectively, and no significant difference was observed between the two groups (*p* = 0.199) (Fig. 1). In the Cox proportional hazards analysis, 75 years of age or older, the occurrence of MACE within the past year, and six or more concomitant drugs taken orally were associated with an increased risk of 5-year MACE compared to the references (adjusted hazard ratios 1.42; 95% Confidence Intervals [CI]: 1.10–1.83; *p* = 0.007, 0.72; 95% CI: 0.55–0.93; *p* = 0.011, and 1.36; 95% CI: 1.01–1.83; *p* = 0.044, respectively) (Table 3).

**Table 3:** Univariate and multivariate analyses to evaluate the association between baseline characteristics and MACE outcomes

Univariate analyses			Multivariate analysis		
Variables	Hazard ratio (95% CI)	*p-*value	Variables	Hazard ratio (95% CI)	*p-*value
Male Sex	1.11 (0.86–1.42)	0.420	Male Sex	1.12 (0.87–1.45)	0.392
75 years of age or older	1.38 (1.08–1.76)	0.011^*^	75 years of age or older	1.42 (1.10–1.83)	0.007^*^
Occurrence of MACE within the past year	0.80 (0.62–1.02)	0.071	Occurrence of MACE within the past year	0.72 (0.55–0.93)	0.011^*^
Concomitant administration of antidiabetic drugs within the past year	1.17 (0.88–1.56)	0.282	Concomitant administration of antidiabetic drugs within the past year	1.03 (0.76–1.40)	0.860
Concomitant administration of antihyperlipidemic drugs within the past year	1.37 (1.07–1.76)	0.014^*^	Concomitant administration of antihyperlipidemic drugs within the past year	1.22 (0.92–1.63)	0.174
Concomitant administration of antiplatelets within the past year	1.03 (0.80–1.31)	0.840	Concomitant administration of antiplatelets within the past year	0.89 (0.67–1.17)	0.389
6 or more concomitant drugs taken orally	1.39 (1.09–1.78)	0.009^*^	6 or more concomitant drugs taken orally	1.36 (1.01–1.83)	0.044^*^
Good medication adherence (PDC ≥ 0.8)	0.69 (0.38–1.23)	0.202	Good medication adherence	0.57 (0.32–1.03)	0.062

* Asterisks indicate significance at the 0.05% level

PDC was estimated at the time of SPC prescription of antihypertensive drugs

Abbreviations: MACE, major adverse cardiovascular events; CI, confidence interval; PDC, proportion of days covered; SPC, single-pill combination

### 5-year follow-up of PDC

At the time of SPC prescription of antihypertensive drugs, the percentages of patients with the PDC within the past year of less than 0.8 and 0.8 or higher were 4.0% [28/707] and 96.0% [679/707], respectively: the PDC values in these two groups were 0.70 ± 0.09 (mean ± standard deviation) and 1.00 ± 0.08, respectively. In the 28 patients in the poor adherence group, the percentages of patients whose PDCs were less than 0.8 in one, two, three, four and five years after SPC prescription was 7.1% [2/28], 17.4% [4/23], 11.1% [2/18], 13.3% [2/15], and 18.2% [2/11], respectively. Among 679 patients in the good adherence group, the percentage of patients with PDCs ≥ 0.8 was 97.1% [659/679] at 1 year, 96.8% [486/502] at 2 year, 96.6% [393/407] at 3 year, 97.0% [326/336] at 4 year, and 95.8% [274/286] at 5 year after the SPC prescription.

## Discussion

In the present study, PDC was calculated for drugs with antihypertensive effects and compared before and after starting an SPC. The results showed no significant difference in PDC between the one-year periods before and after the initiation of the SPC, and the initiation of combination tablets did not lead to an improvement in PDC. A five-year follow-up was also conducted, including censored cases; patients with good adherence prior to the initiation of SPC treatment maintained a relatively high level of medication adherence after starting of SPC treatment. In contrast, among patients with poor adherence, a certain number of them continued to exhibit low adherence after the initiation of SPC treatment. These results suggest that the introduction of SPC treatment did not necessarily contribute to improved medication adherence, although the power to detect a statistically significant difference was not fully sufficient.

The relationship between medication adherence and the occurrence of MACE was not fully understood. Among patients with cardiovascular disease, approximately one year follow-up studies have demonstrated that improved medication adherence is associated with reductions in blood pressure and cholesterol levels, which are identified as risk factors for MACE [9]. More recently, systematic review and meta-analysis have shown that patients with a gradual decline in medication adherence have a 32% higher incidence of MACE [15]. Concerning medication adherence, many subsequent studies evaluating medication adherence have used the traditional 80% threshold as the standard [16–19]; this value was also used in the present study. In the present study, 96.0% (697/707) of the patients had a PDC >0.8 before starting the SPCs, which is considered extremely high, indicating good medication adherence. As stated in the results, the difference in PDC values between the good and poor adherence groups was large relative to their respective standard deviations, and the statistical power was approximately one regardless of the sample size. These results suggest that the PDC was significantly higher in the good adherence group. The NCVC is a hospital in which patients in secondary and tertiary emergency care are accepted; therefore, the proportion of outpatients with low disease severity tends to be lower. It may be said that the higher medication adherence is associated with the high level of awareness of the patients about the need to take medications.

Meanwhile, the use of PDC as an indicator of medication adherence may lead to an overestimation of medication adherence. In general, medication adherence is assessed either directly or indirectly. Direct assessment involves measuring drug levels or biomarkers in blood or urine, providing objective evidence of medication intake. In contrast, indirect assessment includes patient self-reports, pill counts, pharmacy refill records, and electronic monitoring, which estimate medication adherence based on patient behavior or prescription data. In large-scale epidemiological and real-world data studies, the indirect assessment of medication adherence based on PDC calculated from electronic medical records is considered one of the recommended approaches. However, in the present study, whether patients actually took their medications was not directly observed, and the use of PDC as a measure of medication adherence may have contributed to the lack of a significant difference in the occurrence of MACE between the good and poor adherence groups. As shown in Table 2, more than half of the patients had a history of MACE within one year prior to starting SPC. Patients with a history of MACE would be more aware of the importance of blood pressure control with medication, fully understand the significance of taking and staying on medication, and have higher medication adherence. In the present study, the actual medication status of each patient could not be confirmed, although the present data can provide insights into whether the medications were available for the patient. It is considered that patients who can be managed with SPCs are in a relatively stable condition [20]. Since all patients included in this study initiated antihypertensive therapy with SPCs, they were considered to have achieved stable blood pressure control. However, because most of the patients had already demonstrated good medication adherence before the initiation of the SPC treatment, it remains unclear to what extent medication adherence contributed to blood pressure control. Meanwhile, many of them still develop MACE, including recurrences; thus, further information about the factors influencing the development of MACE is needed. Meanwhile, patients with chronic diseases are likely to undergo drug treatment for a long time, and it is considered that they will have difficulty receiving treatment in the event of a disaster; therefore, backup medications are often prescribed. This may cause the PDC to be higher due to the longer prescription days compared to the period of outpatient visits, leading to an overestimation of the PDC value and, thereby, medication adherence.

The incidence of MACE varied widely among studies. In a study in India and Europe, 5% of participants with high cardiovascular risk experienced serious adverse events or cardiovascular events as the first event in the fixed-dose combination group for blood pressure, cholesterol, and platelet control during a follow-up of 12 to 48 months [9]. The use of fixed-dose combinations improves self-reported adherence vs. usual care (86% vs. 65%). The 497 participants at high risk of cardiovascular disease in New Zealand exhibited cardiovascular events, including coronary heart disease events, heart failure hospitalizations, cerebrovascular disease events, and peripheral vascular disease events, at frequencies of 6.3% in the fixed-dose combination treatment using aspirin, statins, and two blood pressure-lowering agents during a median follow-up of 23 months [11]. Self-reported adherence to all these four recommended drugs at 12 months was greater in participants randomized to fixed dose combination treatment compared with usual care (81% vs. 46%). The incidence of MACE in Taiwanese patients newly diagnosed with hypertension who had at least 1 year follow-up was 6.0% in the fixed-dose combination of renin-angiotensin system inhibitors and thiazide diuretics group [7]. The use of fixed-dose combination was associated with better medication adherence (PDC 58%) compared with the free combination group (PDC 47%). In the present study, 86 (12.2%) of the 707 patients experienced MACE during the year follow-up period, which was relatively higher than that reported in previous studies. As mentioned above, the NCVC accepts patients with severe heart disease, which may be the reason why MACE are frequent.

In the present study, SPCs including antihypertensive drugs were analyzed. The administration of two or more antihypertensive drugs in a single pill is considered useful from the viewpoints of both efficacy and safety, probably because multiple antihypertensive drugs with different mechanisms of action in a single pill reduce the physical burden on patients taking these drugs, thereby improving medication adherence. Additionally, improved medication adherence can contribute to stable blood pressure control, which can reduce the risk of cardiovascular events. On the other hand, the medications for treating chronic diseases are not limited to antihypertensive drugs, but also include antidiabetic drugs, statins, antiplatelets, and combinations of these. Differences in the type of SPCs may be associated with differences in the incidence of MACE, although the present study could not analyze all types of SPCs in the market. The number of patients included in the analysis varied among different SPCs, and conducting subgroup analyses based on different SPCs would increase the imbalance in sample sizes. Consequently, the generalizability of the present findings is limited.

In recent years, evidence has accumulated that the risk factors for cerebrovascular diseases other than blood pressure include age, sex, smoking, diabetes, dyslipidemia, chronic kidney disease, and obesity, which have also been introduced in The Japanese Society of Hypertension Guidelines for the Management of Hypertension [20]. In the present study, baseline characteristics such as sex, older age, history of MACE, concomitant drugs (antidiabetic drugs, antihyperlipidemic drugs, and antiplatelet drugs) within the past year, polypharmacy, and good medication adherence were applied in Cox proportional hazards models to estimate their associations with MACE while accounting for the measured confounders. The results showed that 75 years of age or older, occurrence of MACE within the past year, and polypharmacy were significant factors influencing the occurrence of MACE after the initiation of SPC-based antihypertensive therapy (Table 3), which are mostly in line with the results of previous reports. Herein, the finding that a history of MACE was identified as a risk factor for MACE after starting SPC appears to be contrary to clinical intuition: a history of MACE increases the risk of subsequent cerebrocardiovascular events. Clinical guidelines for the management of patients with a history of MACE define target blood pressure levels and recommend evidence-based antihypertensive therapy aimed at minimizing the risk of subsequent events [21–24]. Accordingly, it is considered that blood pressure control achieved through additional antihypertensive therapy following the occurrence of MACE, as well as treatments for comorbid conditions, contributed to the prevention of recurrence; however, detailed analyses of these factors were not conducted in the present study. Polypharmacy is associated with poor adherence to antihypertensive treatment [25], and our present findings showed that poor medication adherence tended to increase the risk of MACE occurrence, but the power to detect the significance was not insufficient.

The present study had some limitations. (1) This was a retrospective cohort study based on a database of single-center sites. Medical records were reviewed to check the history of antidiabetic and antihyperlipidemic drugs for the year prior to the start of the SPC prescription; however, the onset of diabetes or dyslipidemia after the start of the SPC prescription was not examined. Therefore, the extent to which conditions that could have caused the onset of these diseases contributed to the MACE incidence rate remains unclear. In addition to these two diseases, changes in patients’ symptoms during the observation period after prescribing SPCs have not been fully examined, and further investigation is required. (2) Medication adherence does not reflect the patients’ actual medication status. In the present retrospective cohort studies based on databases, it was difficult to determine whether the prescribed medications were actually taken; therefore, the use of PDC may overestimate or underestimate actual medication adherence. (3) The present study did not examine the extent to which blood pressure was controlled, and blood pressure control before and after the prescription of antihypertensive SPCs is not fully understood. More studies are needed to clarify its association with the occurrence of MACE. (4) The poor adherence group was small, and post hoc power analysis indicated a power of 23%. With the good adherence group fixed at 679 participants, the poor adherence group would need to be increased to approximately 164 participants in order to achieve 80% power to detect a difference of 9.6% in the occurrence of MACE at a two-sided significance level of 5%. It may be considered that the imbalance in sample sizes between the two groups contributed to the low statistical power to detect differences in the MACE rate. Future studies are necessary to be conducted in patient groups with balanced baseline characteristics and using large-scale, multicenter data.

In conclusion, the present findings demonstrated that 75 or elder age, history of MACE, and polypharmacy were significant risk factors for MACE after the prescription of antihypertensive SPC, despite the retrospective cohort study design and single-center database. Good medication adherence is thought to contribute to reducing the incidence of MACE, although the extent to which the initiation of SPCs contributes to improved medication adherence could not be clarified.

## Data Availability

The data that support the findings of this study are not openly available for reasons of sensitivity but are available from the corresponding author upon reasonable request.

## Abbreviations

CI: Confidence Interval
ICD: International Classification of Diseases
MACE: Major Adverse Cardiovascular Events
NCVC: National Cerebral and Cardiovascular Center
PDC: Proportion of days covered
SPC: Single-pill combination
